# Combined Therapy of A_1_AR Agonists and A_2A_AR Antagonists in Neuroinflammation

**DOI:** 10.3390/molecules26041188

**Published:** 2021-02-23

**Authors:** Gabriella Marucci, Diego Dal Ben, Catia Lambertucci, Aleix Martí Navia, Andrea Spinaci, Rosaria Volpini, Michela Buccioni

**Affiliations:** Medicinal Chemistry Unit, School of Pharmacy, University of Camerino, 62032 Camerino, MC, Italy; gabriella.marucci@unicam.it (G.M.); diego.dalben@unicam.it (D.D.B.); catia.lambertucci@unicam.it (C.L.); aleix.martinavia@unicam.it (A.M.N.); andrea.spinaci@unicam.it (A.S.); rosaria.volpini@unicam.it (R.V.)

**Keywords:** A_1_AR agonist, A_2A_AR antagonist, combination therapy, neuroinflammation, cytokine, neuroprotection

## Abstract

Alzheimer’s, Parkinson’s, and multiple sclerosis are neurodegenerative diseases related by neuronal degeneration and death in specific areas of the central nervous system. These pathologies are associated with neuroinflammation, which is involved in disease progression, and halting this process represents a potential therapeutic strategy. Evidence suggests that microglia function is regulated by A_1_ and A_2A_ adenosine receptors (AR), which are considered as neuroprotective and neurodegenerative receptors, respectively. The manuscript’s aim is to elucidate the role of these receptors in neuroinflammation modulation through potent and selective A_1_AR agonists (N^6^-cyclopentyl-2′- or 3′-deoxyadenosine substituted or unsubstituted in 2 position) and A_2A_AR antagonists (9-ethyl-adenine substituted in 8 and/or in 2 position), synthesized in house, using N13 microglial cells. In addition, the combined therapy of A_1_AR agonists and A_2A_AR antagonists to modulate neuroinflammation was evaluated. Results showed that A_1_AR agonists were able, to varying degrees, to prevent the inflammatory effect induced by cytokine cocktail (tumor necrosis factor (TNF)-α, interleukin (IL)-1β, and interferon (IFN)-γ), while A_2A_AR antagonists showed a good ability to counteract neuroinflammation. Moreover, the effect achieved by combining the two most effective compounds (**1** and **6**) in doses previously found to be non-effective was greater than the treatment effect of each of the two compounds used separately at maximal dose.

## 1. Introduction

In recent years, many research efforts have been directed to neuroinflammation and neuroimmunology, since neurodegenerative diseases such as Alzheimer’s disease (AD), Parkinson’s disease (PD), and multiple sclerosis (MS) are commonly associated with neuroinflammation induced by activation of microglial cells. In the brain, microglial cells comprise only 5–10% of the total cell population but play a crucial role in dynamic remodeling of the central nervous system (CNS) [1,2,3,4]. These cells are activated in response to changes in brain homeostasis, acquiring phagocytic properties and the ability to release a number of pro-inflammatory molecules [5]. This situation produces a neuroinflammatory reaction, which leads to aggravate neurodegenerative diseases. In addition, chronic neuroinflammation is also implicated in pathology progression; therefore, blockade of this reaction may be expected to improve the outcome of neurodegenerative diseases. Microglia activation involves many signaling pathways, cytokines, and growth factors. Hence, the discovery of drugs that selectively suppress the deleterious effects of microglial activation without compromising its beneficial functions is of primary importance. In recent years, endogenous nucleotides and adenosine (Ado) have been shown to be key messengers in microglial activation process [6,7]. In particular, Ado has been widely recognized as an inhibitory modulator of the CNS [8]. It participates in many functions such as homeostatic modulator at the synapse level, modulating the neuronal excitability, synaptic plasticity, and release of neurotransmitters and is involved in local inflammatory processes [8,9,10,11,12,13]. In addition, during the inflammatory process, extracellular Ado, a ubiquitous molecule implicated in neuromodulation, reaches high concentrations capable of activating the Ado receptors (ARs) A_1_-, A_2A_-, A_2B_-, and A_3_AR [11].

In particular, A_1_AR and A_2A_AR are primarily involved in neuroinflammation modulation [14]. The A_1_AR is expressed in microglia and plays an important role in microglia activation [15]. Activation of A_1_AR produces both pro-inflammatory and anti-inflammatory responses due to different Ado concentrations [16]. In fact, the activation of A_1_AR by low concentrations of adenosine induces neutrophil chemotaxis and adherence to the endothelium [17]; on the contrary, high concentrations of adenosine induce anti-adhesive effects [18]. A study performed with full agonists, as well as the referent compound CCPA (2-chloro-*N^6^*-cyclopentyladenosine), showed a good effect to activate this receptor, but the full agonist for human therapies is correlated to agonist-induced desensitization on the receptor [19] and a variety of side effects. For this reason, in this study, partial agonists synthesized in house were used, which are derivatives of the reference compound. The partial agonist behavior could be beneficial in the treatment of acute and chronic diseases due to less side effects with respect to the reference compound A_1_AR full agonist [20].

In brain injury models, inflammation induced by A_2_AR activation, as well as secretion of interleukin (IL)-18 mediators such as IL-12, interferon (IFN)-γ, and tumor necrosis factor (TNF)-α, was abolished by A_1_AR activation [21], producing a neuroprotective effect in pathological conditions [22]. Moreover, the A_2A_AR is the most implicated adenosine receptor in neuroinflammation [23]. It is worth noting that A_2A_AR expression in microglia is usually low, but it increases as a result of brain insults. In microglial cells, when A_2A_AR overexpression was activated, cytokine release [24] and change in amoeboid morphology occurred [25]. On the contrary, A_2A_AR antagonists suppress microglia activation [26]. Despite the reference compound ZM241385 (4-(2-[7-Amino-2-(2-furyl)[1,2,4]triazolo[2,3-a][1,3,5]triazin-5-ylamino]ethyl)phenol) being the most active A_2A_AR antagonist and, therefore, a useful tool for characterization of responses mediated by A_2A_AR, it also acts as an A_2B_AR antagonist and blocks the cardioprotective effect of adenosine [27]. Hence, in this study, A_2A_AR antagonists synthetized in house with a high selectivity vs. A_2B_AR were used.

## 2. Results

The involvement of A_1_AR and A_2A_AR in neuroinflammation was investigated in the microglial cell line N13 since it is a very interesting model, as the cells behave in the CNS parenchyma as macrophages and are involved in many different neurodegenerative disease processes and traumatic brain and spinal cord injuries.

**Figure 1:**
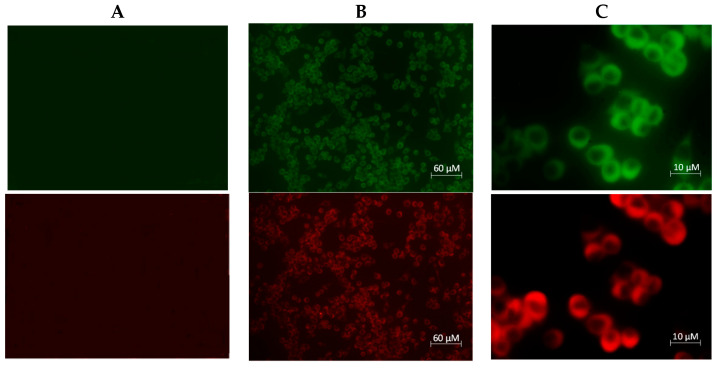
Double immunofluorescence staining of N13 cell cultures. Cells were treated with A_1_ adenosine receptor (AR) polyclonal antibody Alexa Fluor^®^ 488-conjugated (green) and adenosine A_2A_ receptor antibody Alexa Fluor^®^ 594-conjugated (red). (**A**) Negative staining performed in Chinese Hamster Ovary (CHO) wild-type (WT) cells not expressing the adenosine receptors; (**B**) staining of A_1_- and A_2A_AR with Alexa Fluor antibodies (10× magnification); (**C**) staining of A_1_- and A_2A_AR with Alexa Fluor antibodies (60× magnification).

The presence of A_1_AR and A_2A_AR was identified by direct immunocytochemistry using specific polyclonal antibodies (pAbs). The results showed that the amount of receptors was enough to proceed with biological studies to elucidate the relationship between these two receptors and the neuroinflammation process. Experiments were carried out using Alexa Fluor conjugated pAbs and results are shown in Figure 1.

These results obtained in N13 cells, supported by Borea’s and Luongo’s investigations [15,28], allowed to study ligand effects in cell viability. The study was carried out using the CellTiter 96^®^ AQueous One Solution Cell Proliferation Assay. According to the K_i_ value obtained in binding assays, three different concentrations (Table 1) for each compound were tested at 15 and 30 min of incubation. CCPA and ZM241385 were chosen as the reference compounds for the A_1_- and A_2A_ARs, respectively. Each ligand (**1**–**6**) was tested at three different concentrations according to the K_i_ values obtained in the binding assays. The concentrations used were around their K_i_ value at 15 and 30 min incubation time (Table 1 and Table 2).

Since these compounds are known, the binding results are already published [30,31].

The concentrations used for A_1_AR agonists were as follows: compound **1**—4, 8, and 50 μM; compounds **2** and **3**—0.05, 0.1, and 0.6 μM, in comparison with CCPA at 0.005, 0.01, and 0.06 μM (Table 3). In addition, the concentrations studied for A_2A_AR antagonists were as follows: compound **4**—0.2, 0.45, and 2.7 μM; compound **5**—0.02, 0.04, and 0.25 μM; compound **6**—0.15, 0.3, and 2 μM, in comparison with ZM241385 at 0.005, 0.01, and 0.06 μM (Table 3).

The concentrations of the reference compounds were the same, since CCPA for A_1_AR and ZM241385 for A_2A_AR showed identical K_i_ values (1.2 nM).

**Figure 2:**
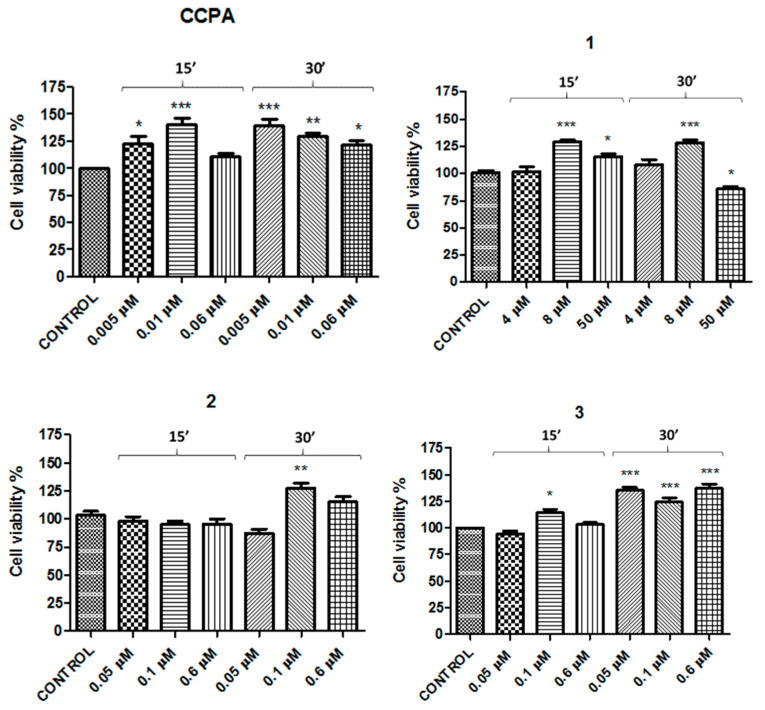
Effects of CCPA and compounds **1**–**3** on N13 cells. Percentage of cell viability after treatment with different ligand concentrations for 15 or 30 min of incubation. Results represent the average of 3–5 independent experiments. * *p* < 0.05, ** *p* < 0.01, *** *p* < 0.001 (one-way ANOVA followed by Dunnett’s multiple comparison test of treated cells against control).

**Figure 3:**
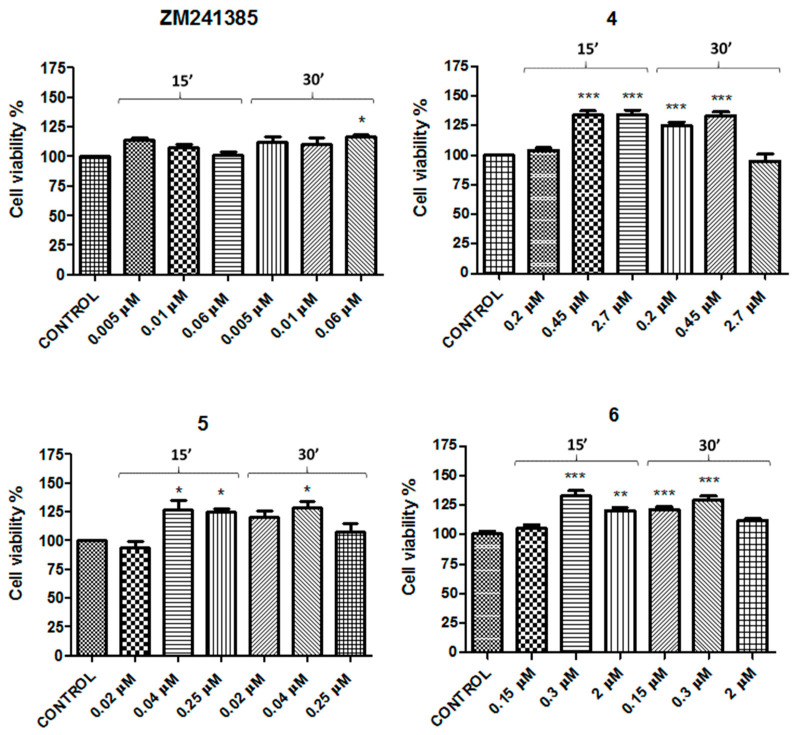
Effects of ZM241385 and compounds **4**–**6** on N13 cells. Percentage of cell viability after treatment with different ligand concentrations for 15 or 30 min of incubation. Results represent the average of 3–5 independent experiments. * *p* < 0.05, ** *p* < 0.01, *** *p* < 0.001 (one-way ANOVA followed by Dunnett’s multiple comparison test of treated cells against control).

The results showed that cell viability was increased with respect to the control for both CCPA and ZM241385 at all concentrations. The highest effect for both was observed at 0.005 μM (139% ± 4.6 and 119% ± 3.4 vs. control, respectively) after 30 min of incubation (Figure 2 and Figure 3).

Compounds **1**, **2**, and **3** also exhibited good viability results at all concentrations used, with the exception of compounds **1** and **2** at the highest and the lowest concentrations of 50 and 0.05 μM after 30 min of incubation, respectively, in which there was a decrease in cell viability with respect to the control (Figure 2).

The A_2A_AR antagonists (**4**–**6**) also appeared to increase the cell viability with respect to the control at all concentrations tested. It is important to note that compounds **4** and **6** increased cell viability in a significative manner, in particular at 0.45 and 0.3 μM at 30 min, respectively (133% ± 5.5 and 129% ± 3.5 vs. control, respectively) (Figure 3).

**Figure 4:**
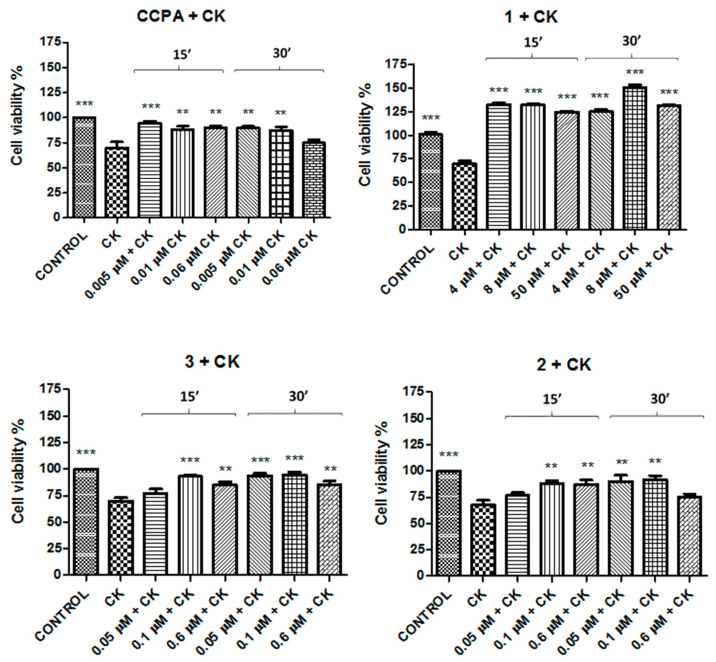
Protective effects of CCPA and compounds **1**–**3** against cytokine (CK) aggression on N13 cells. Percentage of cell viability after treatment with the CK cocktail for 48 h. Results represent the average of 3–5 independent experiments. ** *p* < 0.01, *** *p* < 0.001 (one-way ANOVA followed by Dunnett’s multiple comparison test of treated cells against CK).

In order to verify if these compounds could provide protection in an in vitro inflammatory model, the N13 cell cultures were exposed to a pro-inflammatory cocktail of cytokines (CK) constituted by TNF-α, IL-1β, and IFN-γ 20 ng/mL for 48 h. The results showed that this inflammatory insult decreased cell viability in a significative mode (35% ± 3 vs. control). The experiments in the presence of compounds **1**–**3** and CCPA were performed by pretreating cells with these compounds at the same concentration utilized in precedent experiments (Table 3), 30 min before adding the CK cocktail for 48 h. Results reported in Figure 4 demonstrate that these compounds were able to prevent the damage induced by the CK cocktail as well as CCPA (Figure 4). The best effect was shown by compound **1** at 8 μM after 30 min of incubation (149% ± 3.5 vs. CK).

**Figure 5:**
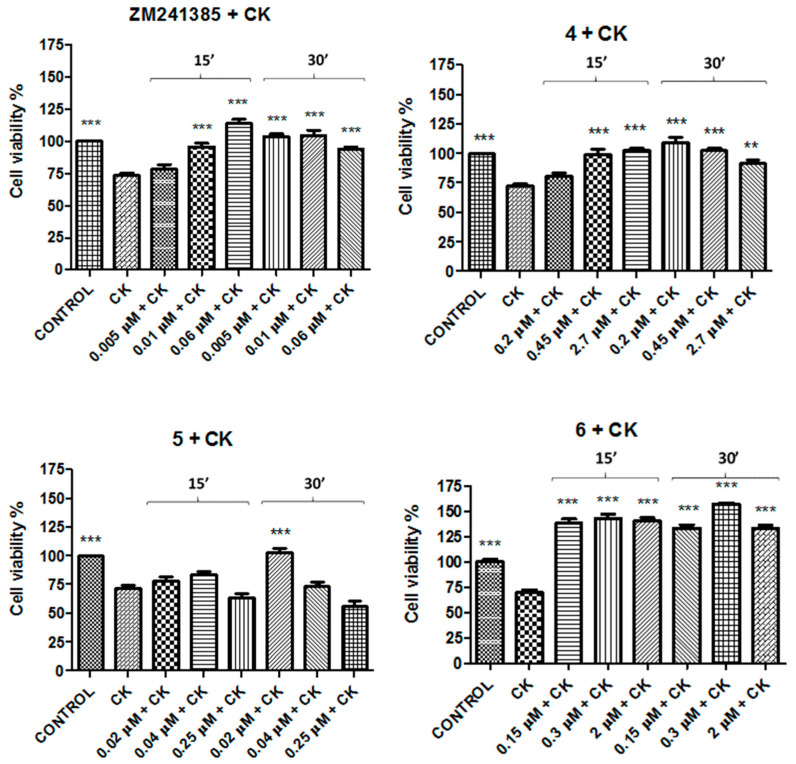
Restoring effects of ZM241385 and compounds **4**–**6** against CK aggression on N13 cells. Results represent the average of 3–5 independent experiments. ** *p* < 0.01, *** *p* < 0.001 (one-way ANOVA followed by Dunnett’s multiple comparison test of treated cells against CK).

In addition, since, in neuroinflammation, there is an overexpression of A_2A_AR, the experiments with A_2A_AR antagonists were performed by pretreating cells with 20 ng/mL of CK cocktail for 48 h and then with compounds **4**–**6** or the referent compound ZM241385 at the same concentration used in cell viability for 15, 30 min (Table 3). The results reported in Figure 5 exhibit that the best effect was obtained by compound **6** at 0.3 μM after 30 min of incubation (157 % ± 3.2 vs. CK).

**Figure 6:**
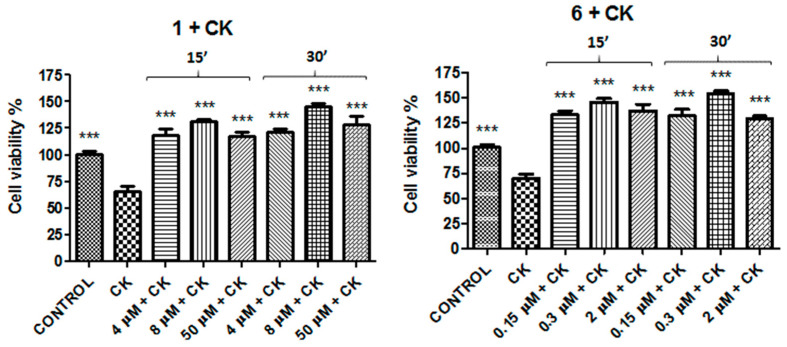
Protective (compound **1**) or restoring (compound **6**) effects after or before incubation of cells with CK. Bar graphs presents the percentage cell viability, and results are presented as the average of 3–5 independent experiments. *** *p* < 0.001 (one-way ANOVA followed by Dunnett’s multiple comparison test of treated cells against CK).

To confirm the results obtained, the two most active compounds (**1** and **6**) were studied using a second cell viability assay based on the quantification of ATP produced by live cells. Experiments were performed according to the procedure carried out with CellTiter 96^®^ AQueous One Solution Cell Proliferation Assay. It was observed that the effects obtained by compounds **1** and **6** were comparable with those detected in the other cell viability assay at 8 and 0.3 μM after 30 min of incubation (145% ± 4.2 vs. CK and 152% ± 4.6 vs. CK, respectively, Figure 6).

**Figure 7:**
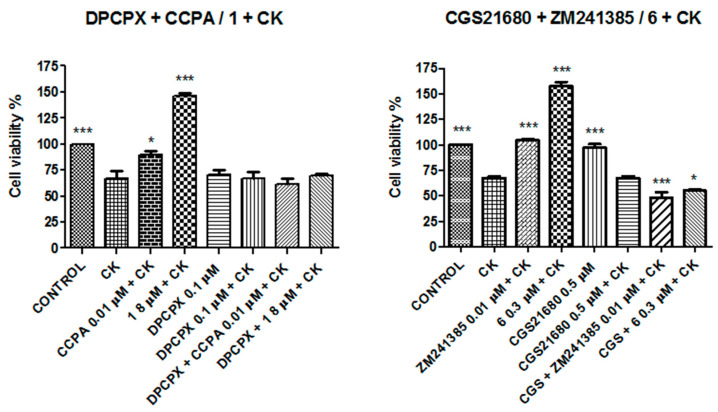
Co-incubation of N13 cells with the A_1_AR antagonist DPCPX and the selective A_2A_AR agonist CGS21680 in combination with compounds **1** and **6** or CCPA and ZM241385, respectively. * *p* < 0.05, *** *p* < 0.001 (one-way ANOVA followed by Dunnett’s multiple comparison test of treated cells against CK).

Since compounds **1** and **6** were shown to be the best compounds to prevent or counteract the effect of CK cocktail, parallel experiments were performed treating, for 30 min, N13 cells with a selective A_1_AR antagonist, DPCPX (1,3-Dipropyl-8-cyclopentylxanthine), and a selective A_2A_AR agonist, CGS21680 (2-(4-(2-Carboxyethyl)phenethylamino)-5′-*N*-ethylcarboxamidoadenosine), in combination with compounds **1** and **6** or reference compounds CCPA and ZM241385, respectively, in order to verify the involvement of these receptors in obtained results. The results are reported in Figure 7.

**Figure 8:**
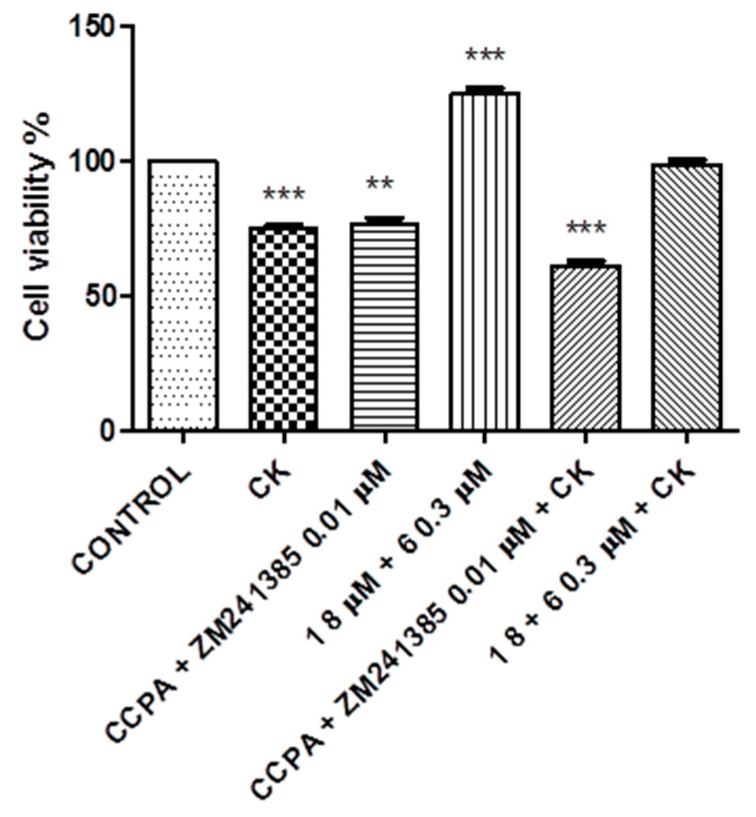
Co-incubation of N13 cells with compounds **1** and **6** or with reference compounds CCPA and ZM241385. Results represent the average of 3–5 independent experiments. ** *p* < 0.01, *** *p* < 0.001 (one-way ANOVA followed by Dunnett’s multiple comparison test of treated cells against control).

At this point, a further study was performed to elucidate a possible synergic effect between compounds **1** and **6** and reference compounds CCPA and ZM241385 to contrast the damage produced by the CK cocktail. Experiments in combination were performed using 8 μM for compound **1,** 0.3 μM for compound **6,** and 0.01 μM for CCPA and ZM241385 after 30 min of incubation. The same experiments were also performed in the presence of the CK cocktail (Figure 8).

**Figure 9:**
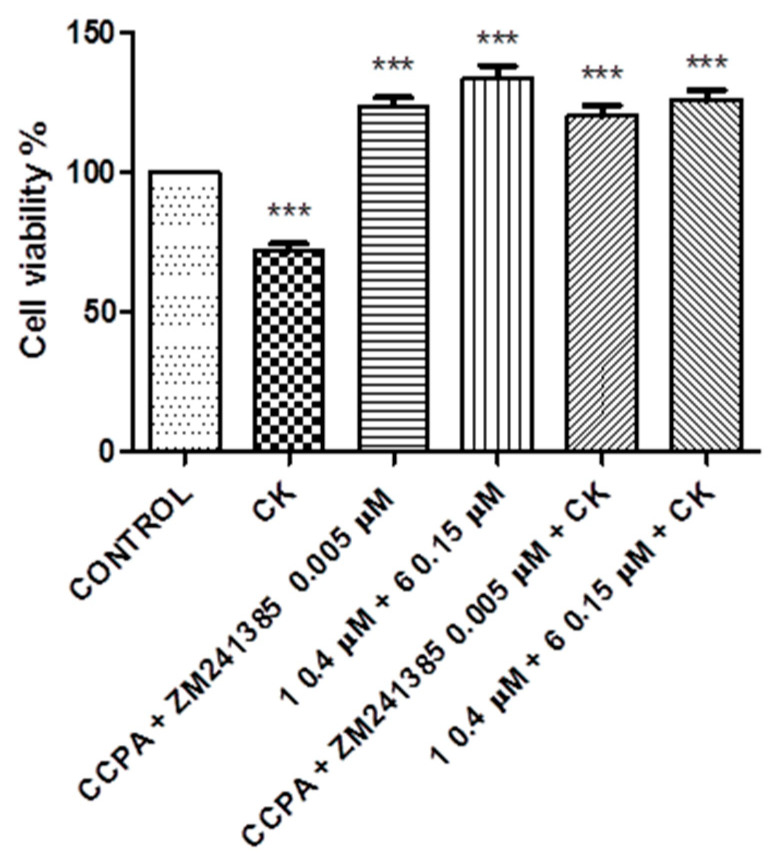
Co-incubation of N13 cells with compounds **1** and **6** or with reference compounds CCPA and ZM241385. Results represent the average of 3–5 independent experiments. *** *p* < 0.001 (one-way ANOVA followed by Dunnett’s multiple comparison test of treated cells against control).

In addition, the same experiment was repeated at lower doses—compound **1** at 0.4 μM and compound **6** at 0.15 μM. The results are reported in Figure 9.

**Figure 10:**
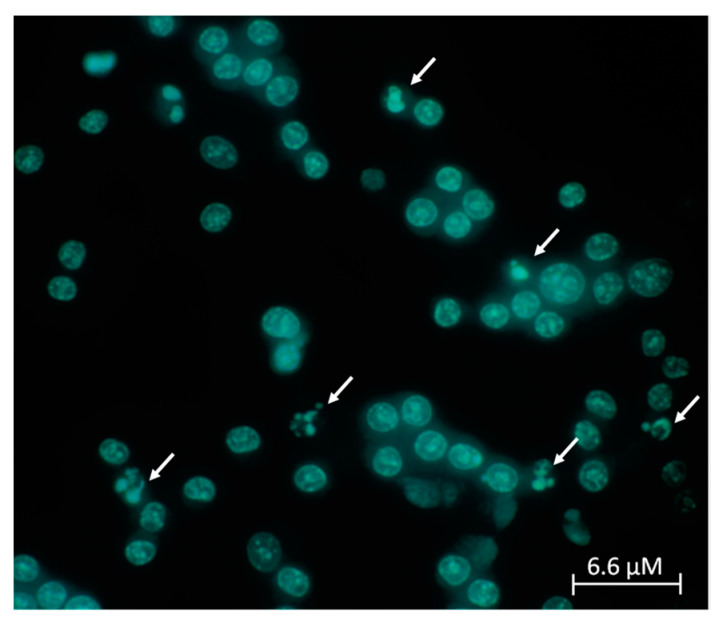
Example of an image obtained through Hoechst assay. In the picture, arrows point to apoptotic cells (40× magnification).

**Figure 11:**
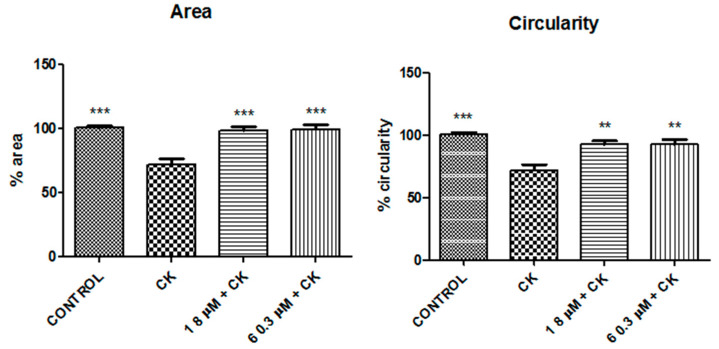
Cell area and circularity after the pre- and post-treatment with compound **1** and compound **6,** respectively, in presence of the CK cocktail. Results represent the average of three independent experiments. ** *p* < 0.01, *** *p* < 0.001 (one-way ANOVA followed by Dunnett’s multiple comparison test of treated cells against CK).

A Hoechst assay was performed to verify the anti-apoptotic properties of compounds **1** and **6**. Image analysis revealed a significant decrease in cell circularity and area detected in cells treated with the CK cocktail compared to control cells (*p*-values < 0.05) (Figure 10 and Figure 11). This decrease was assumed to indicate DNA degradation. The results correlate positively and significantly with those obtained by cell viability assay (R = 0.989, *p*-value < 0.05).

Effects obtained with compounds **1** and **6** after 30 min of incubation indicated that they have a protective effect and contrast the CK aggression in a statistically significant way, demonstrating that they have anti-apoptotic properties (Figure 11).

**Figure 12:**
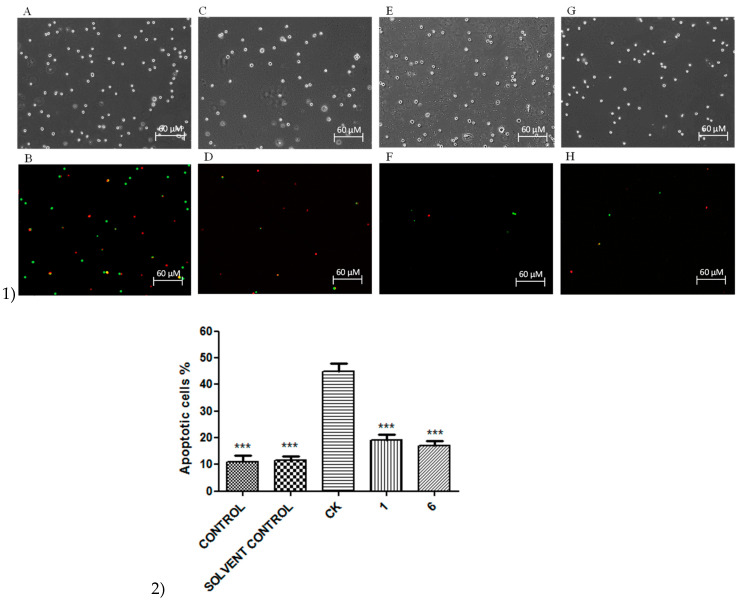
(**1**) Annexin V/PI double staining assay of N13 cells treated for 48 h with CK cocktail (**A**,**B**) and CK cocktail plus compound **1** (**C**,**D**) in comparison with untreated control (**E**,**F**) and solvent control (**G**,**H**). Each group of pictures (10× magnification) is composed of contrast phase and florescence imagines, which underline the anti-apoptotic effect of compound **1**. (**2**) Percentage of apoptotic cells after treatment with CK cocktail or CK plus compound **1** or **6,** in comparison with the untreated control and solvent control. Each bar represents mean ± S.E. *** *p* < 0.001 (one-way ANOVA followed by Dunnett’s multiple comparison test of treated cells or controls against CK).

To confirm the anti-apoptotic properties of compounds **1** and **6**, the Annexin V/PI test was performed. The percentage of apoptotic cells was calculated based on the results of cellular staining. Apoptotic cells were Annexin V-FITC-positive/PI-negative and both Annexin V-FITC/PI-positive. They appeared green (Annexin V-FITC staining), while necrotic cells appeared red (PI staining) and apo-necrotic cells light orange (Figure 12). Compounds **1** and **6** were incubated at concentrations of 8 and 0.3 μM, respectively. Generally, the anti-apoptotic effect was observed with both compounds and the percentages of apoptotic cells were similar. In Figure 12 (panel 1) are reported the results obtained with compound **1** as example. The percentage of apoptotic cells treated with compounds **1** and **6** was 19% and 21%, respectively, followed by 45% in CK cocktail treatment and 11% and 12% in untreated cells and cells treated with solvent, respectively (Figure 12 panel 2).

## 3. Discussion

The present study provides evidence for the involvement of A_1_AR and A_2A_AR in neuroinflammation regulation. Neuroinflammation is a pathological state of inflammatory responses localized in the brain and spinal cord. Since chronic neuroinflammation is correlated to neurodegeneration, there is a significant interest in understanding the mechanisms involved in inflammatory responses [32]. Microglia are the principal cells involved in innate immune surveillance and their activation is key in neuroinflammation. These myeloid cells express ARs and it is well known that total expression of A_1_AR and A_2A_AR changes after aggression. Indeed, there are many research findings [33,34,35] that have reported the changes in ARs; in particular, A_1_AR is downregulated and A_2A_AR upregulated after an insult. This work was carried out starting from this evidence using full and partial agonists to stimulate the A_1_AR and antagonists to block the A_2A_AR in in vitro models of neuroinflammation. Immunostaining experiments were performed in order to verify the presence of A_1_AR and A_2A_AR in N13 cells, which are essential to exclude false negative and/or positive results. The presence of A_1_AR and A_2A_AR was confirmed by the double immunofluorescence staining of N13 cell cultures in the presence of adenosine A_1_ receptor polyclonal antibody Alexa Fluor^®^ 488-conjugated and adenosine A_2A_ receptor antibody Alexa Fluor^®^ 594-conjugated. A pilot cell viability experiment was performed up to 72 h, but since there was no difference with respect to 30 min, the experimental time choice was 30 min. The results of cell viability proved that the reference compounds CCPA and ZM241385 were able to increase cell viability with respect to the control, and the maximum effect was observed at 0.005 μM (139% ± 4.6 and 119% ± 3.4 vs. control, respectively) after 30 min of incubation (Figure 2 and Figure 3). All of the A_1_AR agonists and A_2A_AR antagonists exhibited very similar results (Figure 2 and Figure 3), demonstrating a non-toxic effect. Experiments carried out up to 72 h showed only a non-significant decrease (*p* < 0.05) in cell viability, confirming a non-toxic profile also after a long exposure.

Activated microglia release some cyto-/chemokine-based signals, especially during emergencies, representing a rich and instant brain endogenous source for numerous cyto- and chemokines. Cytokines constitute a significant portion of the immuno- and neuro-modulatory messengers that can be released during microglia activation. However, excessive or sustained activation significantly contributes to acute and chronic neuropathologies. Dysregulation of microglial cytokine production promotes harmful actions of the defense mechanisms, resulting in direct neurotoxicity. For these reasons, the experiments were performed in presence of a CK cocktail in order to reproduce the induced neurotoxicity. These experiments were performed taking into account the different expression levels of A_1_AR and A_2A_AR during neuroinflammatory pathologies. The decrease in A_1_AR in microglia, macrophages, and neurons leads to a state of neuroinflammation and the activation of A_1_AR leads to neuroprotective effects [36,37,38,39,40]. On the other hand, overexpression of A_2A_AR is associated to chronic stress and A_2A_AR stimulation is involved in neurodegeneration [41,42]. On these bases, the experiments with A_1_AR agonists were performed using compounds before the insult with CKs, and they were present during and after the inflammatory insults for the entire experiment duration. On the contrary, A_2A_AR antagonists were administrated after the aggression with the CK cocktail in order to decrease the upregulation induced. The reported results demonstrate that this strategy is accurate; in fact, the administration of agonists before the CK insult completely prevented the aggression and the antagonists after CK counteracted the damage effect of them as well as CCPA and ZM241385, respectively. The same experiments were performed with another cell viability assay based on the quantification of ATP produced by live cells to corroborate the results. The best results, in both cell viability assays, were obtained with the agonist compound **1** at 8 μM and the antagonist compound **6** at 0.03 μM, both after 30 min of incubation. For this reason, these two compounds were chosen for further experiments. It is worthwhile to note that compound **1** is a partial A_1_AR agonist and this produces a desired submaximal response, avoiding the overstimulation of receptors [43]. Furthermore, note that desensitization of G protein-coupled receptors (GPCRs), such as A_1_AR, is activated almost simultaneously with their activation. Partial agonists produce agonist-dependent receptor phosphorylation decreased by protein-coupled receptor kinase G (GRK) with a reduction in desensitization consequent to a prolongation of activation and fewer side effects [44].

After the inflammatory insult, a significant increase in cell viability was observed both with compounds **1** and **6**. The treatment with compound **1** at 8 μM for 30 min produced an increase in cell viability of 149%. A similar result was observed with compound **6** at 0.3 μM (157 %) for 30 min of incubation (Figure 3 and Figure 4). The effect of the cell viability increase produced by compounds **1** and **6**, alone and in the presence of the CK insult, after 30 min is imputable both to increased cell proliferation and to an increase in cellular metabolic processes. This assertion was confirmed by results obtained with trypan blue experiments that allowed quantifying the cell proliferation.

The experiments performed with DPCPX, a selective A_1_AR antagonist, and CGS21680, a selective A_2A_AR agonist, in combination with compounds **1** and **6** or with reference compounds CCPA and ZM241385 demonstrated that the addition of DPCPX produces cell damage similarly to the CK cocktail, and co-incubation with compound **1** or CCPA did not ameliorate the cell viability. In fact, it is clear that DPCPX antagonized the effects of both CCPA and compound **1** since, in its presence, there was not a recovery in decreased cell survival produced by the CKs (150%). On the other hand, incubation with the A_2A_R agonist CGS21680 did not produce a decrease in cell viability, but at the same time, it did not counteract the effect of the CK cocktail. In addition, co-incubation with CGS21680 and ZM241385 or compound **6** did not produce any increase in cell viability with respect to the CK cocktail. In conclusion, these experiments confirmed the involvement of A_1_AR and A_2A_AR in the effects produced by compounds **1** and **6**, respectively.

These results underline the ability of these compounds, by A_1_AR and A_2A_AR interaction, to prevent, in vitro, microglial death and promote microglial growth. Further studies are planned to verify if these actions will be confirmed in vivo.

The best efficacy is often obtained with combination therapies that produce a decrease in toxicity and a reduced development of drug resistance. Combination therapies have become a standard for the treatment of several diseases, continuing to feature a promising approach in indications where a single treatment is doomed to failure [45,46,47,48]. In this context, since the A_1_AR agonist and A_2A_AR antagonist counteract the neuroinflammation produced by a CK cocktail, co-administration of these ligands could bring an increased benefit against it. The experiment performed using a combination with compounds **1** and **6** and the compounds CCPA and ZM241385 showed that there was not an additive effect when administering couples of compounds in combination, but it is interesting to note that compounds **1** and **6** were demonstrated to be much more effective in counteracting CK damage than the reference compounds (Figure 8). This result is probably imputable to the dose effect-based approaches; in fact, the dose that produced the maximum effect of each compound was used. For this reason, further experiments were performed with a combination of sub-threshold doses. The non-effective doses of compounds **1** and **6** or CCPA and ZM241385 produced a significant (*p* < 0.005) increased effect in combination compared to the maximum dose of each compound (Figure 9). Compounds **1** and **6** demonstrated, in this case also, to be more effective in counteracting CK damage than the reference compounds. This finding provides new opportunities for the rational development of combination therapies in neuroinflammation, limiting side effects.

In recent years, there have been many papers regarding tests about the study of the well-known A_1_AR agonist CCPA and A_2A_AR antagonists SCH 58261 (2-(2-Furanyl)-7-(2-phenylethyl)-*7H*-pyrazolo[4,3-e][1,2,4]triazolo[1,5-c]pyrimidin-5-amine) and ZM241385 in neurodegenerative disorders, both in cells and animal models. On the contrary, until now, there is no evidence about the use of A_1_AR partial agonists and A_2A_AR antagonists in therapeutic approaches used in combination. Since an emerging anti-neurodegenerative role for prolonged A_1_AR activation and A_2A_AR inhibition could represent a targeted strategy in multiple neurodegenerative diseases such as Alzheimer’s, Parkinson’s, and multiple sclerosis, the combination of A_1_AR partial agonists and A_2A_AR antagonists represents a future prospect for the treatment of these pathologies [35,49,50,51,52,53,54,55].

In recent years, various studies demonstrated that the secretion of cytokines by microglia activation induces the activation of two distinct pathways, apoptosis and nuclear factor kappa-light-chain-enhancer of activated B cells (NF-κB) [49,56]. In order to verify the anaptotic effect of compounds **1** and **6,** two experiments, a Hoechst assay and an Annexin V-FITC test, were performed.

The Hoechst assay was performed in order to verify if the cell circularity and area were influenced by the CK cocktail and by compounds **1** and **6**. After treatment with the CK cocktail, there was a significant decrease in cell circularity and area, and this indicates possible DNA degradation, but after treatment with compounds **1** and **6,** there was a protective effect and a contrast to CK aggression in a statistically significant way, demonstrating the anti-apoptotic properties of these compounds (Figure 11). Further confirmation was obtained by the Annexin V-FITC test in combination with propidium iodide that is able to differentiate between apoptosis and necrosis. In fact, the Annexin V permitted the identification of viable, transient apoptotic and necrotic cells, since cells undergoing apoptosis or necrosis have clearly distinct morphological features. In the early apoptosis stages, exposure to phosphatidylserine (PS) on cell surface occurs, followed by membrane blebbing, nuclear fragmentation, decreased cell volume, and formation of apoptotic bodies. On the other hand, programmed necrosis morphological features include early plasma membrane rupture and rapid cytoplasmic and nuclear swelling [57]. For these reasons, apoptotic-like cell death is often quantified by measuring PS externalization by binding of Annexin V.

Apoptotic Annexin V-positive cells were confirmed by microscopical analysis in comparison to untreated N13 cells. These studies provide evidence that chromatin condensation coincides with exposure to CK cocktail. In fact, as shown in Figure 12A,B, after CK cocktail exposure, it is possible to evaluate, by Annexin V/PI staining, double-positive late apoptotic cells and necrotic cells in an amount of 45% in CK cocktail treatment, and 11% in untreated cells (Figure 12G,H). On the other hand, in the presence of compounds **1** and **6,** the amount of double-positive late apoptotic cells and necrotic cells was greatly reduced (Figure 12C–F) to a percentage of 19% and 21%, respectively. This experiment confirmed the results obtained with the Hoechst assay, demonstrating that compounds **1** and **6** possess good anti-apoptotic properties.

## 4. Materials and Methods

Compounds **1**–**6** were synthesized at the School of Pharmacy of Camerino University (Italy) and dissolved in dimethyl sulfoxide (DMSO) to prepare a 10-mM stock solution, which was then diluted with water to the concentration required for the experiments. In all experiments, the maximum concentration of DMSO in wells did not exceed 0.5% and had no effect on cell viability. CCPA, ZM241385, CGS21680, DPCPX, and Hoechst 33258 were purchased from Sigma-Aldrich (Milan, Italy). All materials concerning cell culture were purchased from EuroClone S.p.A. (Milan, Italy); CellTiter 96^®^ AQueous One Solution Cell Proliferation and CellTiter-Glo^®^ Luminescent Cell Viability Assays were purchased from Promega (Milan, Italy). The Annexin V-FITC Apoptosis Detection Kit (BioVision) was bought from DBA S.r.L. (Milan, Italy).

### 4.1. Cells Culture

N13 cells were grown in DMEM (Dulbecco’s Modified Eagle Medium) High Glucose supplemented with 10% fetal bovine serum, 100 U/mL penicillin, 100 µg/mL streptomycin, 1 mM sodium pyruvate, and 2 mM l-glutamine in a humidified atmosphere with 5% CO_2_ at 37 °C. Compounds were dissolved in DMSO to have a final concentration of 10 mM and, prior to use, were then diluted with water.

### 4.2. Immunocytochemistry

A_1_AR and A_2A_AR presence was investigated using an immunofluorescence technique. A_1_AR presence was checked using the adenosine A_1_ receptor polyclonal antibody Alexa Fluor^®^ 488-conjugated, while A_2A_AR was studied by adenosine A_2A_ receptor antibody Alexa Fluor^®^ 594-conjugated. Briefly, media were aspired and cells fixed with Fixative Solution for 15 min (high-purity 4% formaldehyde in PBS (Phosphate Buffered Saline), pH = 7.3). After that, they were washed 3 times with PBS and permeabilized with a Permeabilization Solution (0.5% Triton X-100) for 15 min [58]. They were washed again with PBS and incubated for 1 h with Blocking Buffer (3% BSA (Bovine Serum Albumin), fraction V delipidated in PBS). Finally, antibody labeling proceeded and cells were seeded in a 6-well plate at 3 × 10^5^ cells per well.

### 4.3. Trypan Blue Exclusion Test

Briefly, 3 × 10^5^ N13 cells were seeded in a six-well plate and incubated for 24 h. Subsequently, compounds **1** and **6** were added at a concentration of 8 and 0.3 μM, respectively, in the absence or presence of a CK cocktail. After the incubation, cells were detached with Trypsin-EDTA solution and centrifuged at 1200 rpm for 10 min. Cell viability was determined using trypan blue staining, using a Countess II Automated Cell Counter (Thermo Fisher Scientific, USA) to count the number of live and dead cells.

### 4.4. Cell Titer 96^®^ AQueous One Solution Cell Proliferation Assay

Briefly, 1 × 10^4^ N13 cells were plated in 98 μL medium and incubated in a 96-well plate overnight. After 24 h, 2 μL ligand or CK cocktail was added to the wells. After treatment, 20 µL CellTiter 96^®^ AQueous One Solution Reagent was added in each well and allowed to incubate for 1 h [59]. Absorbance was measured at 492 nm in a microplate reader. Cell viability was calculated as a percentage using the formula: (mean OD (Optical Density) of treated cells/mean OD of control cells) × 100. Results were expressed as percentage of control cells that were not treated. An untreated control and a control with the solvent were run. All experiments were performed in triplicate.

### 4.5. CellTiter-Glo^®^ Luminescent Cell Viability Assay

Briefly, 1 × 10^4^ N13 cells were seeded into 96-well cell culture plates and incubated overnight. After 24 h, 2 μL ligand or CK cocktail was added to the wells. After treatment, cells were lysed using CellTiter Glo reagent, and the luminescence signals produced by ATP molecules from metabolically active cells were measured using a plate reader after 30 min incubation at room temperature. Results were expressed as percentage of control cells that were not treated. An untreated control and a control with the solvent were run. All experiments were performed in triplicate.

### 4.6. Hoechst Assay

A Hoechst assay was carried out to check the anti-apoptotic effect of the understudy compounds using Hoechst 33258. Briefly, 3 × 10^5^ N13 cells were seeded in a six-well plate. After 24 h, the media were eliminated and cells washed with PBS. Then, they were washed with acetic acid/methanol solution 50:50, washed again with PBS, and incubated for 10 min with a fixative solution. After this, cells were cleaned with distilled water and incubated light-protected for 30 min at rt with Hoechst (1 µg/mL). Finally, the dye was discarded and cells were washed with water. Glycerol solution was added and cells were observed under an Olympus microscope. The cell area and circularity were measured using ImageJ as the image analyzing software [60]. Area and circularity were calculated as percentages using the following formula:

$$\mathrm{Area}\;\mathrm{or}\;\mathrm{circularity}\;=\frac{\mathrm{area}\;\mathrm{or}\;\mathrm{circularity}\;\mathrm{of}\;\mathrm{treated}\;\mathrm{cells}}{\mathrm{area}\;\mathrm{or}\;\mathrm{circularity}\;\mathrm{of}\;\mathrm{control}\;\mathrm{cells}}\times 100$$

### 4.7. Apoptosis and Necrosis Assay

The Annexin V-FITC test was performed to evaluate the anti-apoptotic effect of understudy compounds thanks to the capability of the kit to differentiate between apoptosis and necrosis. Briefly, 3 × 10^5^ N13 cells were seeded in a six-well plate. After 24 h, the media were eliminated and cells were washed with PBS and centrifuged. The pellet was resuspended with 500 µL binding buffer and 5 µL Annexin V-FITC and 5 µL propidium iodide were added. The mix was incubated at room temperature for 5 min in the dark. Cell suspension was added on a glass slide and covered with a glass coverslip, and then, the glass slide was monitored with a fluorescence microscope coupled to a cell imaging system, using a dual filter set for FITC and rhodamine [61]. Approximately 300 cells were used for each analysis. Results are the mean of three different experiments.

### 4.8. Statistical Analysis

Quantitative data are presented as means ± SE from 3–5 independent experiments. The significance of differences was evaluated using a two-tailed Student’s *t*-test or one-way ANOVA followed by Dunnett’s post-test. Statistical analysis was carried out with GraphPad Prism8 Software (San Diego, CA, USA). *p* ≤ 0.05 were considered as statistically significant.

## 5. Conclusions

Existing evidence indicates that chronic inflammation mediated by the activation of microglia plays a significant role in neurodegenerative diseases. A_1_AR is considered a neuroprotective receptor, and A_2A_AR is designated as a neurodegenerative receptor; therefore, A_1_AR stimulation and A_2A_AR inhibition could be one of the most promising strategies to treat neurodegenerative diseases. The aim of this work was to elucidate the role of these receptors in neuroinflammation modulation using potent and selective A_1_AR agonists and A_2A_AR antagonists. Among the studied ligands, compounds **1** and **6** were found to be particularly active in counteracting CK damage, even more than the reference compounds, and it was proven that the effect exerted was due to the interaction with A_1_AR and A_2A_AR. Moreover, since the two compounds have shown a synergistic effect in counteracting the apoptotic effect induced by CK, they could represent a promising approach for the therapy of neurodegenerative diseases in which a single treatment is doomed to failure.

## Figures and Tables

**Table 1:**
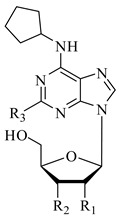
Biological activity of A_1_AR compounds studied and reference ligand at human (h)ARs ^a^ [19,20].

Compound	R_1_	R_2_	R_3_	hA_1_ ^b^ K_i_ nM	hA_2A_ ^c^ K_i_ nM	hA_2B_ ^e^ EC_50_ nM	hA_3_ ^d^ K_i_ nM
**1**	H	OH	H	816	>30,000	>30,000	>30,000
**2**	OH	H	H	10.5 (±1.8)	16,000 (±714)	>30,000	7190 (±510)
**3**	OH	H	Cl	10.4 (±2)	10,400 (±806)	>30,000	5300 (±1081)
CCPA	OH	OH	Cl	1.2 (±0.2)	2050 (±400)	18,800 (±320)	26 (±5)

^a^ Data (*n* = 3−5) are expressed as means ± standard errors. ^b^ Displacement of specific [^3^H]-CCPA binding at hA_1_AR expressed in CHO cells. ^c^ Displacement of specific [^3^H]-NECA binding at hA_2A_AR expressed in CHO cells. ^d^ Displacement of specific [^3^H]-HEMADO binding at hA_3_AR expressed in CHO cells. ^e^ EC_50_ value (nM) of adenylyl cyclase activity stimulation in CHO cells expressing hA_2B_AR.

**Table 2:**
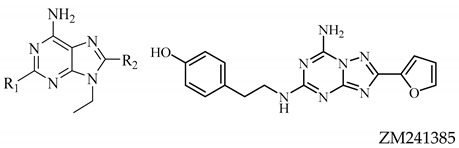
Biological activity of A_2A_AR compounds studied and reference ligand at human (h)ARs ^a^ [19,20].

Compound	R_1_	R_2_	hA_1_ ^b^ K_i_ nM	hA_2A_ ^c^ K_i_ nM	hA_2B_ ^e^ IC_50_ * K_i_ nM	hA_3_ ^d^ K_i_ nM
**4**	H	OCH_2_CH_3_	752 (±188)	45 (±13)	7940 (±1588)	>30,000
**5**	H	2-Furyl	49 (±9)	2.6 (±0.4)	1000 (±100)	4700 (±890)
**6**	OCH_2_CH_3_	OCH_2_CH_3_	4247 (±842)	32 (±6.3)	4860 (±516)	948 (±187)
ZM241385	−	−	255 (±35)	1 (±0.18)	* 50 (±0.11)	>30,000

^a^ Data (*n* = 3−5) are expressed as means ± standard errors. ^b^ Displacement of specific [^3^H]-CCPA binding at hA_1_AR expressed in CHO cells. ^c^ Displacement of specific [^3^H]-NECA binding at hA_2A_AR expressed in CHO cells. ^d^ Displacement of specific [^3^H]-HEMADO binding at hA_3_AR expressed in CHO cells. ^e^ IC_50_ values of NECA-stimulated adenylyl cyclase activity inhibition in CHO cells expressing hA_2B_AR. * Ongini et al. 1999 [29].

**Table 3:** A. Concentrations of A_1_AR ligands tested.

Compound	Concentration 1 (μM)	Concentration 2 (μM)	Concentration 3 (μM)
**CCPA**	0.005	0.01	0.06
**1**	4	8	50
**2**	0.05	0.1	0.6
**3**	0.05	0.1	0.6
B. Concentrations of A_2A_AR ligands tested			
**ZM241385**	0.005	0.01	0.06
**4**	0.2	0.45	2.7
**5**	0.02	0.04	0.25
**6**	0.15	0.3	2

## Data Availability

No new data were created or analyzed in this study.

## Sample Availability

Samples of the compounds are available from the authors.
